# Focusing on Cracks with Instance Normalization Wavelet Layer

**DOI:** 10.3390/s25010146

**Published:** 2024-12-29

**Authors:** Lei Guo, Fengguang Xiong, Yaming Cao, Hongxin Xue, Lei Cui, Xie Han

**Affiliations:** 1 Shanxi Key Laboratory of Machine Vision and Virtual Reality, North University of China, Taiyuan 030051, China; hopenxfg@nuc.edu.cn (F.X.); yafei4554@foxmail.com (Y.C.); xhxchj2279@nuc.edu.cn (H.X.); hanxie@nuc.edu.cn (X.H.); 2 Shanxi Province’s Vision Information Processing and Intelligent Robot Engineering Research Center, North University of China, Taiyuan 030051, China; 3 School of Computer Science and Technology, North University of China, Taiyuan 030051, China; 4 National Supercomputer Center, Shandong Computer Science Center, Jinan 250013, China; alencui@outlook.com

**Keywords:** crack detection, wavelet, convolution neural networks, feature fusion

## Abstract

Automatic crack detection is challenging, owing to the complex and thin topologies, diversity, and background noises of cracks. Inspired by the wavelet theory, we present an instance normalization wavelet (INW) layer and embed the layer into the deep model for segmentation. The proposed layer employs prior knowledge in the wavelets to capture the crack features and filter the high-frequency noises simultaneously, accelerating the convergence of model training. Furthermore, instance normalization in our layer is utilized to mitigate the feature differences, boosting the generalization capability. In addition, a fusion layer is added to merge the information across the different layers. The comparison experiments and ablation studies demonstrate that the INW layer steadily enhances recognition and convergence performance on the DeepCrack dataset and CRACK500 dataset.

## 1. Introduction

Concrete crack, one of the most common engineering defects, directly impacts the structural integrity of the building and is therefore a crucial and early indicator of concrete health assessment [1]. Crack detection is of great significance in reducing maintenance charges and mitigating further severe damage. Currently, manual crack detection is the dominant but time-consuming approach. Regarding crack detection, it is tough to identify thin and illegible cracks, whereas detecting the type of crack maintains a high input–output ratio. Consequently, effective and efficient crack detection becomes an urgent need, especially for difficult-to-detect cracks.

Crack detection has been extensively studied over the past decades. Traditional crack detection methods include the threshold segmentation approach, region-growing approach, and traditional machine learning-based approach [2]. The threshold segmentation approach works by separating the foreground and background based on a predefined threshold. However, this approach is highly sensitive to threshold values and is not well suited for crack detection in complex backgrounds. The region-growing approach offers some resistance to noise, but it requires considerable time to set seed pixels, making it less suitable for real-time detection. The traditional machine learning-based approach, on the other hand, can exhibit some degree of adaptability, often combined with feature extraction techniques to achieve high-precision crack detection. However, the approach struggles to effectively model complex data and tends to have weak generalization capabilities [3]. Today, deep learning-based methods are the main methods of crack detection. Having sufficient data and employing end-to-end learning can enhance the networks’ ability to learn robust and efficient representations. Typically, crack detection is treated as the task of image segmentation. These methods adopt an encoder–decoder architecture to fulfill crack detection, e.g., Unet [4,5], FCN [6], and the corresponding variants [7,8,9]. The encoder extracts the global and local features by stacking multiple standard convolution layers, and the decoder outputs the high-level semantic information by upsampling the features. In this process, in order to preserve the details of the image, the decoder also employs low-level features to achieve high-precision segmentation, achieving excellent results in crack detection. However, when faced with crack detection under complex environments, the performance of the above methods declines, owing to the diverging and thin local structures of the cracks, and the strong background noise. These approaches adopt standard convolutions initialized by conventional strategies that cannot extract effective features from thin cracks. Furthermore, the amount of crack data is small, and the samples vary greatly, leading to inefficient optimization.

**Figure 1:**
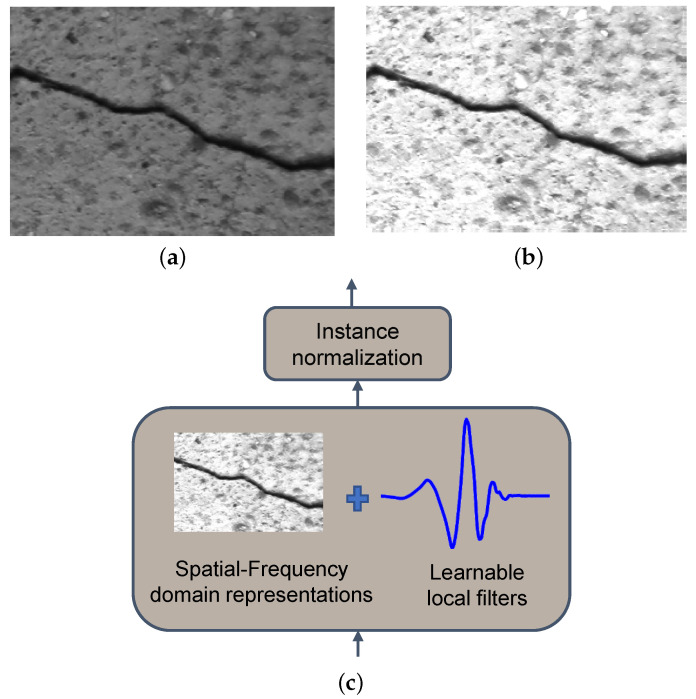
An illustration of (**a**) A Grayscale image, (**b**) the corresponding low-frequency wavelet features, and (**c**) the instance normalization wavelet layer.

In this paper, we propose a simple yet efficient module, namely, the instance normalization wavelet (INW) layer, for crack detection. Differing from the Fourier transform the wavelet transform, as a spatial–frequency analysis tool, it captures the thin mutations effectively. The wavelet transform calculates the inner products between the employed wavelet functions and the signals. As shown in Figure 1, by 2D wavelet transform for the crack images, the low-frequency component retains the vast majority of details for the cracks and suppresses the background noises significantly. From a signal-processing perspective, convolution layers can be viewed as data-driven adaptive wavelet filters which extract features in the horizontal and vertical directions. Inspired by this idea, we embed the wavelet transform with a priori knowledge into the deep learning-based detection model, aiming to strengthen the representation learning ability for these subtle cracks. Compared with the standard wavelet transform, the corresponding weights are learned in an end-to-end way. Additionally, we add instance normalization after the wavelet transform to alleviate the differences in the features of the cracks. These strategies enable the adaptability and generalization of our method. Our architecture is largely based on BiSeNet V2 [10], which is a representative image semantic segmentation architecture. The architecture consists of two complementary branches, the detail branch and the semantic branch. The first branch is used to extract detailed information, and the second branch is employed to extract global information. To boost the ability of local feature extraction, we primarily made modifications in the detail branch. We incorporate two serial INW layers at the low level and one INW layer at the middle level, respectively. Deformable convolution is employed to expand the receptive fields in the first layer. In addition, we leverage a convolution layer to fuse the local and global predictions of these two branches. Extensive experiments on DeepCrack and CRACK500 datasets demonstrate that our method outperforms the recent crack detection methods.

To summarize, the contributions can be listed as follows:

1. We propose a framework for crack detection. Our framework is proficient at capturing the thin features, owing to the INW layer, deformable convolution layer and, and fusion layer.
2. We design the INW layer, motivated by the wavelet transform mechanism. Based on the corresponding a priori knowledge, we calculate the inner products between the adaptive wavelets and the features and normalize the representation, refining and denoising the features.
3. Comprehensive experiments verify the performance of the presented framework on the aspects of detection and convergence. The ablation studies demonstrate the effectiveness of the designed module.

The rest of this paper is organized as follows. Section 2 summarizes the related work from the aspects of crack detection and wavelet transform in vision. Section 3 introduces the proposed method. The implementation details and experimental results are presented in Section 4, followed by conclusions and future work in Section 5.

## 2. Related Work

### 2.1. Crack Detection

Automatic crack detection, as a form of non-destructive testing (NDT), offers significant advantages in terms of reducing labor costs while maintaining high diagnostic accuracy. From an application standpoint, crack detection is predominantly employed in the assessment of pavements. These pavements include buildings (roads, bridges, and walls), steel, and leather [11]. While these materials vary considerably in terms of their properties, the methods employed for crack detection exhibit a high degree of applicability and reference value. Automatic crack detection can be categorized into two types: traditional methods and deep learning-based methods. Traditional methods employ threshold-based strategy to detect cracks. Kamaliardakani et al. [12] uniformized the background and developed a heuristic thresholding approach. Zhang et al. [13] presented an adaptive thresholding segmentation method considering spatial and geometric features. Aiming to enhance the adaptability of crack detection methods to complex samples, researchers have proposed a series of strategies. In the modeling process, a crack probability map is constructed to strengthen the connection of the predictions [14]. In [15], Salman et al. employed the Gabor Filter to extract features, retaining the detailed information. In [16], wavelet transform is employed to remove noises and to enhance the edges of cracks. Meanwhile, the effectiveness of conventional filters for detection was revealed in [15,16]. Nonetheless, the traditional methods still suffer from vulnerability to the complex environment, owing to the limited modeling capacity. Recently, deep learning has dominated computer vision tasks and has also been applied in the domain of crack detection. These works used advanced net architectures, e.g., Fully Convolutional Networks (FCNs) and Unet, and their variants to fulfill the segmentation task. Compared with the high-level computer vision tasks, the detailed features are of greater significance in crack detection. Liu et al. [6] employed FCN as the backbone and fused the multi-level features. Pyramidal feature representations were extracted by Feature Pyramid Networks to boost accuracy and generalizability [17]. In [4], Unet was used for crack detection, and the impact of the background was investigated. Cui et al. [18] also utilized Unet and proposed an attention gate module to focus attention on the key areas. Furthermore, recent works adapted to the domain of crack detection by refining the feature extraction modules. Zhou et al. [19] adopted mixed pooling rather than spatial pooling to maintain the low-level sharp information. Dilated convolution with a large receptive field was applied to extract vision features with irregular topologies [7]. Space-to-Depth Conv was employed in the CrackTinyNet to prevent the excessive loss of tiny object information, rather than traditional downsampling [20]. Wang et al. used a transformer-based architecture to capture the long-range interactions and obtain better representation [21]. Nonetheless, it is still necessary to enhance the model’s feature extraction ability for cracks in the complex environment.

### 2.2. Application of Wavelet Transform in Vision

Wavelet transform is a fundamental mathematical tool for multi-resolution analysis. Compared to the Fourier transform, which struggles with non-stationary signals, the wavelet transform effectively extracts both global and local features by the multiscale basis functions [22,23]. The wavelet transform has been widely applied in various fields, such as signal processing, oil exploration, and material analysis [24,25,26]. And the wavelet transform has also been applied in computer vision tasks for decades [27,28]. With the development of artificial intelligence, wavelet transform is introduced into deep learning to strengthen the representation ability of neural networks. In contrast, the corresponding weights are learned in a data-driven way. Wavelet transform was employed to fuse the RGB and thermal infrared images from a frequency perspective, to reduce the impact of morphological difference [29]. Williams et al. [30] regarded wavelet transform as downsampling to correct issues such as edge halos and blurring caused by deterministic pooling. The most related works are [31,32]. These works proposed two wavelet-based attention mechanisms that implemented the feature enhancement across all frequency bands. For crack detection, the low-frequency component retains the most information of the cracks and is reserved for the computation afterward, compared with the other three components [33]. In this way, denoising is completed for the backgrounds simultaneously. And the instance normalization is inserted to alleviate the distribution shift, focus on discriminative features, and thereby boost the model’s generalization ability.

## 3. Proposed Method

**Figure 2:**
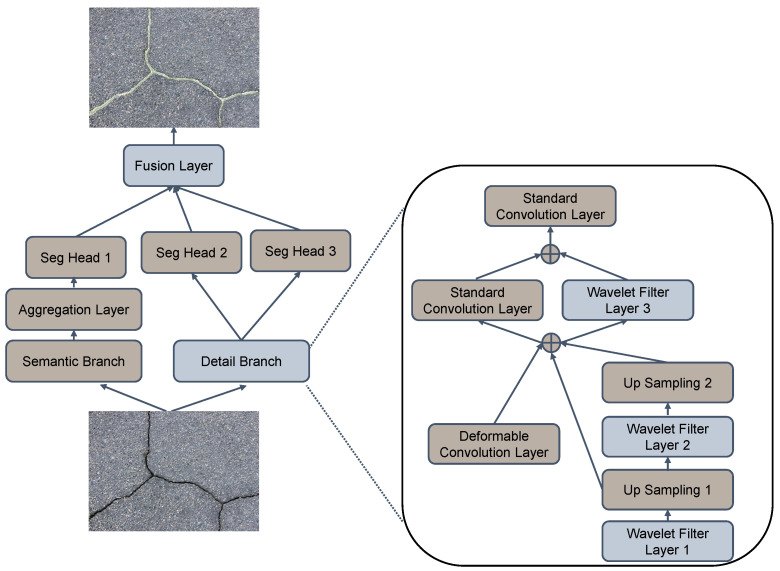
The architecture of the proposed INWB network.

The architecture of the INW bilateral network (INWB) is shown in Figure 2. Cracks are usually subtle with a certain level of background noises. And the vision features vary in different materials. These characteristics affect the detection performance critically. Consequently, we propose an INW layer to extract the crack detailed skeleton and eliminate the background noises. In this work, we only retain the low-frequency component, and add instance normalization for the module. Such a design filters the high-frequency background noises elegantly and boosts the generalization ability. Finally, we insert three INW layers in the detail branch, and one fusion layer upon the segmentation heads, formulating the INW bilateral network.

### 3.1. Instance Normalization Wavelet Layer

The challenge of crack detection lies in the background noise and diversity of the samples. Background noises lower the data quality and global contrasts. Spatial filters, e.g., average filtering and Gaussian filter, are effective in reducing the noise amplitude. Unfortunately, the structure of thin cracks will be destroyed in a certain degree. Wavelet analysis extracts features from the perspective of spatial and frequency domains, hence preserving the details. From another perspective, the designed wavelet layer contains prior engineering knowledge, which can endow the neural network with optimal initial weights, thereby facilitating convergence. The diversity of the cracks and backgrounds affects the robustness of the model. To preserve the structure of the crack and boost the generalization ability, we embed the normalized wavelet layer to the network from the spatial–spectral aspect. The corresponding architecture is given in Figure 1. Let $X^{\mathrm{ori}}\in R^{H\times W}$ be the feature map, where *C* is the number of channels, and *H* and *W* are the height and width of the feature map. The wavelet transform utilizes the inner product calculation to match the spatial step signals and is suited for crack detection. We utilize the 2D wavelet transform to extract the spatial–spectral features, and obtain the low-frequency and high-frequency components of images, respectively:

(1) $$X^{LL}\left(m,\;n\right)=\frac{1}{\sqrt{MN}}\sum_{i=1}^{M-1}\sum_{j=1}^{N-1}X^{ori}\left(x,\;y\right)W_{mn}^{LL}\left(x,\;y\right)$$

(2) $$X^{HL}\left(m,\;n\right)=\frac{1}{\sqrt{MN}}\sum_{i=1}^{M-1}\sum_{j=1}^{N-1}X^{ori}\left(x,\;y\right)W_{mn}^{HL}\left(x,\;y\right)$$

(3) $$X^{LH}\left(m,\;n\right)=\frac{1}{\sqrt{MN}}\sum_{i=1}^{M-1}\sum_{j=1}^{N-1}X^{ori}\left(x,\;y\right)W_{mn}^{LH}\left(x,\;y\right)$$

(4) $$X^{HH}\left(m,\;n\right)=\frac{1}{\sqrt{MN}}\sum_{i=1}^{M-1}\sum_{j=1}^{N-1}X^{ori}\left(x,\;y\right)W_{mn}^{HH}\left(x,\;y\right)$$

where $X^{\mathrm{LL}}$ is the low-frequency component; $X^{\mathrm{LH}}$, $X^{\mathrm{HL}}$, and $X^{\mathrm{HH}}$ are the high-frequency components in the horizontal, vertical, and diagonal directions; and $W^{\mathrm{LL}}$, $W^{\mathrm{LH}}$, $W^{\mathrm{HL}}$, and $W^{\mathrm{HH}}$ are the corresponding neural network weights inheriting the weights of the wavelet transform. Note that the weights of the INW layer are not fixed because of the learning process. The initial values of $W_{\mathrm{init}}^{\mathrm{LL}}$, $W_{\mathrm{init}}^{\mathrm{HL}}$, $W_{\mathrm{init}}^{\mathrm{LH}}$, $W_{\mathrm{init}}^{\mathrm{HH}}$ are as follows:

$$W_{\mathrm{init}}^{\mathrm{LL}}=g^{L}\times {\left(g^{L}\right)}^{T}$$

$$W_{\mathrm{init}}^{\mathrm{HL}}=g^{H}\times {\left(g^{L}\right)}^{T}$$

$$W_{\mathrm{init}}^{\mathrm{LH}}=g^{L}\times {\left(g^{H}\right)}^{T}$$

$$W_{\mathrm{init}}^{\mathrm{HH}}=g^{H}\times {\left(g^{H}\right)}^{T}$$

where $g^{L}$ and $g^{H}$ are the coefficients of the low-pass filter and high-pass filter, respectively. The selection of an appropriate wavelet basis is a factor influencing the effectiveness of this method. The symmetry and compact support characteristics of the bior wavelet confer certain advantages in image processing, which we validated in subsequent experiments. The learned weights are specific compared to these in the general wavelet transforms. In the study, we observe that the high-frequency components contain more noise, which has no positive effect on detection. Therefore, we abandon the high-frequency components. Next, instance normalization is utilized to alleviate the discrepancy between samples [34]:

(5) $$X_{IN}^{LL}=\gamma \left(\frac{X^{LL}-\mu }{\sigma }\right)+\beta$$

where

(6) $$\mu =\sum_{i=1}^{U}\sum_{j=1}^{V}X_{ij}^{LL}$$

(7) $$\sigma =\sqrt{\frac{1}{UV}\sum_{i=1}^{U}\sum_{j=1}^{V}\left(X_{ij}^{LL}-\mu \right)+\varepsilon }$$

Unlike batch normalization, instance normalization calculates μ and σ from the sample level and has a stronger generalization ability.

### 3.2. Fusion Layer

In contrast to typical segmentation tasks, crack detection focuses more on details. At the same time, global features are equally important for the task. We argue that the predictions across different layers of the two branches contribute to the prediction. As a result, we add a fusion layer to aggregate the local and global predictions. First, we concatenate the outputs from the detail and semantic branches along the corresponding axis. Second, 1×1 convolutional layer is used to fulfill the final prediction:

(8) $$\mathrm{out}={\mathrm{conv}}_{1\times 1}\left(x\right)$$

where **x** is the concatenated predictions, and **out** is the final output. For the fusion layer, the number of output channels is determined by the number of classes. In this study, the considered classes are crack and background, resulting in an output channel number of 2.

### 3.3. INWB Architecture

The architecture of the INWB adopts the basic structure of BiSeNet V2, and consists of two branches, detail and semantic branches, as shown in Figure 2. The detail branch is employed to extract the high-resolution features, whereas we utilize the semantic branch to obtain the global features with fast downsampling. First, we insert three INW layers to the detail branch and replace standard convolution with deformable convolution. At the beginning, we add the corresponding upsampling modules to maintain dimensions that are consistent with the deformable convolution features. Via such a strategy, the background noises are removed via the data-driven wavelet transform, and thin features are extracted in a lossless way with deformable convolution. Second, we use the convolution layer as the final fusion layer to integrate the predictions of the two branches. Third, we employ the Online Hard Example Mining Cross Entropy [35]. In the method, the threshold is set to mine hard samples and achieve focused training, mitigating the influence of the imbalance between the crack and the background. Furthermore, the training algorithm of INWB is detailed in Algorithm 1.
**Algorithm 1:** The training algorithm of INWB.Inputs: input images **X**, detail(), semantic(), aggregation(), main_head(), aux1_h(), aux2_h(), fusion()Model initialization: Insert three INW layers into the detail branch of BiSeNet V2, and initialize the corresponding parameters with the bior family wavelet basis. Add fusion_layer() to fuse the outputs of main_head(), aux1_head(), and aux2_head().For **x** in **X**:      x1_1=detail(x)      aux1,aux2,x1_2=semantic(x)      output1=aggregation(x1_1,x1_2)      output1=main_head(output1)      aux1,aux2=aux1_h(aux1),aux2_h(aux2)      output=fusion(output,aux1,aux2)      L=OHEM(output,gt)      Update model with adamw based on *L*End**return** the trained model

## 4. Experiments

### 4.1. Datasets and Implementation Details

To reveal the effectiveness of our proposed method, we adopt the DeepCrack dataset [6] and CRACK500 dataset [36] as the benchmarks. The DeepCrack dataset comprises 537 color images, in which 300 images are used for training, and 237 images are employed for verification. There is a noticeable imbalance in the distribution of crack pixels and background pixels, with crack pixels comprising only 3.54% of the total in this dataset. CRACK500 dataset includes 1896 images for training and 1124 images for testing. At first, the color images are converted to grayscale images. During training, we utilize the strategies of adjusting the sharpness and contrast, flip, Gaussian blur, rotation, and crop for data augmentation. The batch size is 6. All the methods are verified on a single NVIDIA RTX 3090 GPU with a memory of 24 G.

### 4.2. Evaluation Metrics

We employ precision, recall, and F-score as the evaluation metrics. Precision measures the accuracy of crack predictions made by the segmentation model. Recall evaluates the ability of a model to recognize all the crack pixels. F-score is a comprehensive metric that balances precision and recall. In this paper, we employ the F-score as the main metric. The equations are as follows:

(9) $$Precision=2\times \frac{TP}{TP+FP}$$

(10) Recall=2×TPTP+FN

(11) $$F-score=2\times \frac{Precision\times Recall}{Precision+Recall}$$

where *TP*, *FP*, and *FN* are the true positives, false positives, and false negatives, respectively.

### 4.3. Ablation Studies

To verify the influences on the performance of each module, we conduct ablation studies from three aspects, containing the impact of the INW structure, feature visualization, and module analysis.

(1) Impact of INW structure

In this subsection, we study the impact of the INW structure. First, we investigate the effect of different-frequency components in Table 1. According to Table 1, employing the HH component results in the lowest mF-score, while incorporating the LH and HL components leads to a slight decline in performance compared to using the LL component alone. We hypothesize that although high-frequency components contain some useful information, the internal noise significantly impacts the final segmentation performance. Therefore, we exclude the LH, HL, and HH components, for the simplicity of the model. Second, we compare four wavelet families including haar, db, rbio, and bior. Table 2 illustrates the results using different wavelet families. The gap for the F-score, the comprehensive index, does not exceed 0.08. The family of bior slightly outperforms the other three families, and bior is used in this paper.

(2) Feature visualization

**Figure 3:**
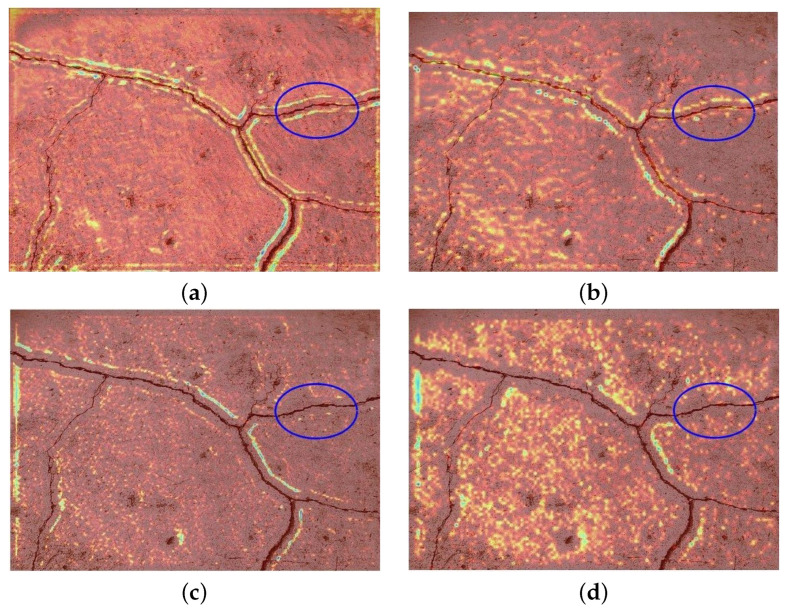
Feature visualization by Grad-CAM: (**a**) low-level features extracted by INW; (**b**) middle-level features extracted by INW; (**c**) low-level features extracted by the baseline; (**d**) middle-level features extracted by the baseline.

We take a deeper look into the models by visualizing the features over different layers in the training process. The corresponding results are summarized in Figure 3. Figure 3a,b show the feature visualization in the first convolution layers after INW modules 2 and 3, and the corresponding feature visualization after removing the INW module is given in Figure 3c,d. The highlighted regions in Figure 3 denote the discriminative areas learned by the models. Comparatively, the features extracted by INW effectively encapsulate the cracks, whereas the baseline method exhibits discontinuities along the cracks, particularly within the elliptical region. Moreover, the highlighted areas in the features extracted by INW are more concentrated, further demonstrating the superior feature extraction capability of INW.

(3) Module analysis

We investigate the contributions of INW and fusion layer to the overall performance, as shown in Table 3. Specifically, the INW module captures the features of the mutation signals, and injects the prior knowledge into the framework; compared with the BiSeNet V2, feature fusion leverages the low-level and high-level features to output detailed crack prediction. In contrast to the baseline, it can be observed that INW and fusion layer achieve improvements of 0.030 and 0.019 on the F-score, respectively. Consequently, the effectiveness of these two modules is verified.

### 4.4. Main Results

To verify the effectiveness of our method, we compare it with the other four typical crack segmentation methods. (1) Unet is one of the most classical binary image segmentation methods of the encoder–decoder architecture, demanding fewer training samples [37]. (2) Deeplabv3plus is another conventional convolution architecture for semantic segmentation, which utilizes the atrous convolution to expand the receptive fields and removes all the max pooling layers to retain the details [38]. For DeepLabv3plus, we utilize two versions. In DeepLabv3plusFree, all pretrained weights are used for training, while in DeepLabv3plusFrozen, the backbone weights are frozen. (3) FCN presents a fully convolutional architecture which enables efficient and accurate pixel-wise predictions [39]. (4) SETR is the early work using the backbone of a vision-transformer (without convolution), modeling the context information [40].

**Figure 4:**
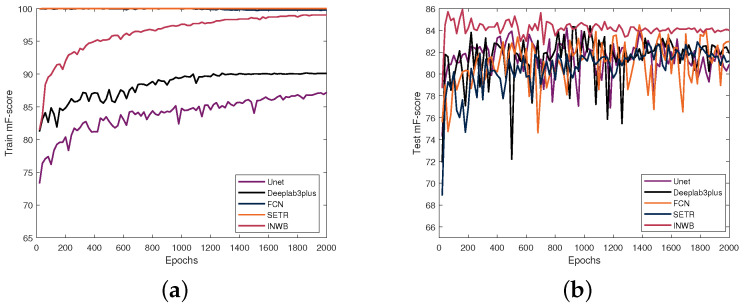
F-score curves on the DeepCrack dataset: (**a**) training dataset; (**b**) test dataset.

The experimental results in the DeepCrack dataset are summarized in Table 4 and Figure 4. First, the detection performance is compared in Table 4. Regarding the detection performance, our method beats other methods. The F-scores of Unet, Deeplabv3plusFree, Deeplabv3plusFrozen, FCN, SETR, and our method are 0.842, 0.844, 0.850, 0.845, 0.830, and 0.859, respectively. The CNN-based methods, i.e., Unet, Deeplabv3plus, FCN and our methods beat the transformer-based methods. It is because the inductive bias in the convolution operation contributes to the detailed feature extraction. In terms of computational complexity, the SETR method is significantly higher than other CNN-based methods. Our method has comparable FLOPs to other CNN-based methods. It is worth noting that DeepLabv3plusFrozen outperforms DeepLabv3plusFree. This may be because the pretrained backbone is already capable of extracting high-quality features, and due to the limited training data, using all weights for training may lead to overfitting. As a result, our method is slightly superior to the other CNN-based methods. Our method achieves better performance due to the INW structure being able to extract more refined local features.

To further evaluate the performance of the proposed method, we conduct validation on the CRACK500 dataset. Compared to the DeepCrack dataset, the CRACK500 dataset features a more diverse background and greater data complexity. The results are given in Table 5. While the precision of other methods exceeds 0.7, our method achieves a precision of 0.693. However, for recall, our method reaches 0.921, outperforming all other methods, which remain below 0.9. Additionally, our method achieves an F-score of 0.791, at least 0.041 higher than that of competing methods. Regarding computational complexity, similar conclusions hold for all the algorithms when evaluated on the CRACK500 dataset as discussed in the DeepCrack dataset. These results demonstrate that our method exhibits superior overall performance on this dataset.

Second, to verify the impact of the proposed module on the convergence performance of the model, we provide performance curves for the training and testing processes as shown in Figure 4. We observe that our method effectively promotes convergence. First, FCN and SETR quickly approach 100% F-score on the training set, while F-scores on the test set can consistently fluctuate significantly, indicating that these two methods have weak generalization performance in situations with limited data. Nonetheless, the F-scores of Unet and Deeplabv3plus are lower on both the training and testing sets, reflecting the relatively low modeling ability of these two methods. Our method exhibits high and stable performance on both the training and testing sets. We argue that it is because the embedding of prior knowledge results in well-initialized weights, leading to fast convergence and good generalization ability.

## 5. Discussion

The effectiveness of the INW layer is thoroughly validated through ablation studies and the main results. By studying the impact of the INW structure, we select an appropriate wavelet family. Feature visualization confirms that INW improves the saliency of discriminative features. Comparison experiments demonstrate the superiority of our method in terms of detection performance and computational complexity. Our approach integrates the wavelet layer into the model, effectively denoising and accelerating convergence, while leveraging instance normalization to reduce the impact of sample variations, thereby enhancing the generalization capability.

## 6. Conclusions

In this paper, we propose a novel crack detection method that integrates an INW layer and a fusion layer into a segmentation network. This approach is designed to address the challenges posed by the diversity of crack patterns, background variations, and background noise. The INW layer is introduced to filter noise and normalize the representation of each sample, guiding the learning process and enhancing the network’s generalization ability. Additionally, a fusion layer aggregates predictions from three segmentation heads at different network levels, effectively leveraging low-level to high-level features to improve detection performance. Comparative experiments and ablation studies have validated the effectiveness of the proposed method. In the future, we will investigate issues such as few-shot learning and extend its evaluation to more crack detection datasets to further validate its generalization ability.

## Figures and Tables

**Table 1:** The experimental results of different-frequency components on the DeepCrack dataset.

Wavelet Component	Precision	Recall	F-Score
LL	0.858	0.860	0.859
HH	0.733	0951	0.801
LL, LH, HL	0.821	0.873	0.846
LL, LH, HL, HH	0.829	0.882	0.855

**Table 2:** The experimental results of wavelet families on the DeepCrack dataset.

Wavelet Family	Precision	Recall	F-Score
haar	0.819	0.887	0.852
db	0.822	0.884	0.852
rbio	0.831	0.878	0.854
bior	0.858	0.860	0.859

**Table 3:** Ablation analysis of INW and fusion layer.

Methods	INW	Fusion Layer	Precision	Recall	F-Score
Baseline	−	−	0.803	0.818	0.810
+INW	✓	−	0.841	0.841	0.840
+Fusion Layer	−	✓	0.770	0.898	0.829
All	✓	✓	0.858	0.860	0.859

**Table 4:** The experimental results of precision, recall, and F-score on DeepCrack dataset.

Methods	Precision	Recall	F-Score	FLOPs
Unet	0.849	0.835	0.842	0.162T
Deeplabv3plusFree	0.880	0.810	0.844	0.141T
Deeplabv3plusFrozen	0.858	0.842	0.850	0.141T
FCN	0.857	0.834	0.845	0.158T
SETR	0.887	0.779	0.830	0.284T
INWB	0.858	0.860	0.859	0.158T

**Table 5:** The experimental results of precision, recall, and F-score on CRACK500 dataset.

Methods	Precision	Recall	F-Score	FLOPs
Unet	0.703	0.673	0.688	0.190T
Deeplabv3plusFree	0.724	0.707	0.715	0.155T
Deeplabv3plusFrozen	0.760	0.741	0.750	0.155T
FCN	0.720	0.733	0.726	0.174T
SETR	0.640	0.693	0.665	0.325T
INWB	0.693	0.921	0.791	0.186T

## Data Availability

The dataset is within the article.
